# Red fluorescent protein-based cAMP indicator applicable to optogenetics and *in vivo* imaging

**DOI:** 10.1038/s41598-017-07820-6

**Published:** 2017-08-04

**Authors:** Kazuki Harada, Motoki Ito, Xiaowen Wang, Mika Tanaka, Devina Wongso, Ayumu Konno, Hirokazu Hirai, Hajime Hirase, Takashi Tsuboi, Tetsuya Kitaguchi

**Affiliations:** 1 Department of Life Sciences, Graduate School of Arts and Sciences, The University of Tokyo, 3-8-1 Komaba, Meguro, Tokyo 153-8902 Japan; 2 Department of Biological Sciences, Graduate School of Science, The University of Tokyo, 7-3-1 Hongo, Bunkyo, Tokyo 113-0033 Japan; 3 Laboratory for Neuron-Glia Circuitry, RIKEN Brain Science Institute, Hirosawa 2-1, Wako-shi, Saitama 351-0198 Japan; 4 Cell Signaling Group, WASEDA Bioscience Research Institute in Singapore (WABIOS), 11 Biopolis Way, #05-02 Helios, Singapore, 138667 Singapore; 5 Department of Neurophysiology and Neural Repair, Gunma University Graduate School of Medicine, Maebashi, Gunma 371-8511 Japan; 6 Comprehensive Research Organization, Waseda University, #304, Block 120-4, 513 Wasedatsurumaki-cho, Shinjuku, Tokyo 162-0041 Japan; 7 Laboratory for Chemistry and Life Science, Institute of Innovative Research, Tokyo Institute of Technology, 4259 Nagatsuta-cho, Midori-ku, Yokohama 226-8503 Japan

## Abstract

cAMP is a common second messenger that is involved in various physiological processes. To expand the colour palette of available cAMP indicators, we developed a red cAMP indicator named “Pink Flamindo” (Pink Fluorescent cAMP indicator). The fluorescence intensity of Pink Flamindo increases 4.2-fold in the presence of a saturating dose of cAMP, with excitation and emission peaks at 567 nm and 590 nm, respectively. Live-cell imaging revealed that Pink Flamindo is effective for monitoring the spatio-temporal dynamics of intracellular cAMP generated by photoactivated adenylyl cyclase in response to blue light, and in dual-colour imaging studies using a green Ca^2+^ indicator (G-GECO). Furthermore, we successfully monitored the elevation of cAMP levels *in vivo* in cerebral cortical astrocytes by two-photon imaging. We propose that Pink Flamindo will facilitate future *in vivo*, optogenetic studies of cell signalling and cAMP dynamics.

## Introduction

Cyclic adenosine monophosphate (cAMP) is an important second messenger that mediates hormone secretion, cell migration and memory formation^1–3^. Although previous studies have developed several Förster resonance energy transfer (FRET)-based cAMP indicators, and clarified the properties of intracellular cAMP dynamics including basal and maximum levels^4–7^, they seldom serve as indicators in multi-colour imaging approaches because the fluorescence must be measured at two different wavelengths. Here we aimed to develop a single fluorescent protein (FP)-based cAMP indicator that requires only one emission to be measured for imaging.

We previously developed green FP-based cAMP indicators called Flamindo and Flamindo2, which we demonstrated could be used to monitor cAMP dynamics simultaneously with Ca^2+^, or exocytosis by dual-colour imaging^8, 9^. However, monitoring cAMP dynamics with green fluorescence restricts the applicability to multi-colour imaging, because there is currently only a limited choice of red or blue fluorescent indicators available for live-cell imaging. In addition, it is difficult to combine optogenetic strategies with blue light-excited fluorescent indicators, such as Flamindo, as many optogenetic tools (including channel rhodopsins and photoactivated adenylyl cyclases (PACs)) are themselves activated by blue light^10, 11^. To overcome these drawbacks, we developed a red FP-based cAMP indicator named Pink Flamindo (Pink Fluorescent cAMP indicator). Pink Flamindo exhibits a 4.2-fold increase in fluorescence intensity upon cAMP binding and our functional analyses of Pink Flamindo demonstrated its utility in dual-colour imaging, optogenetic and *in vivo* imaging approaches in living mice. We consider that Pink Flamindo is a powerful tool to monitor cAMP dynamics in conjunction with other signalling molecules, not only in single cells but also in whole animals during optogenetic stimulation.

## Results and Discussion

### Development of a red cAMP indicator Pink Flamindo

**Figure 1:**
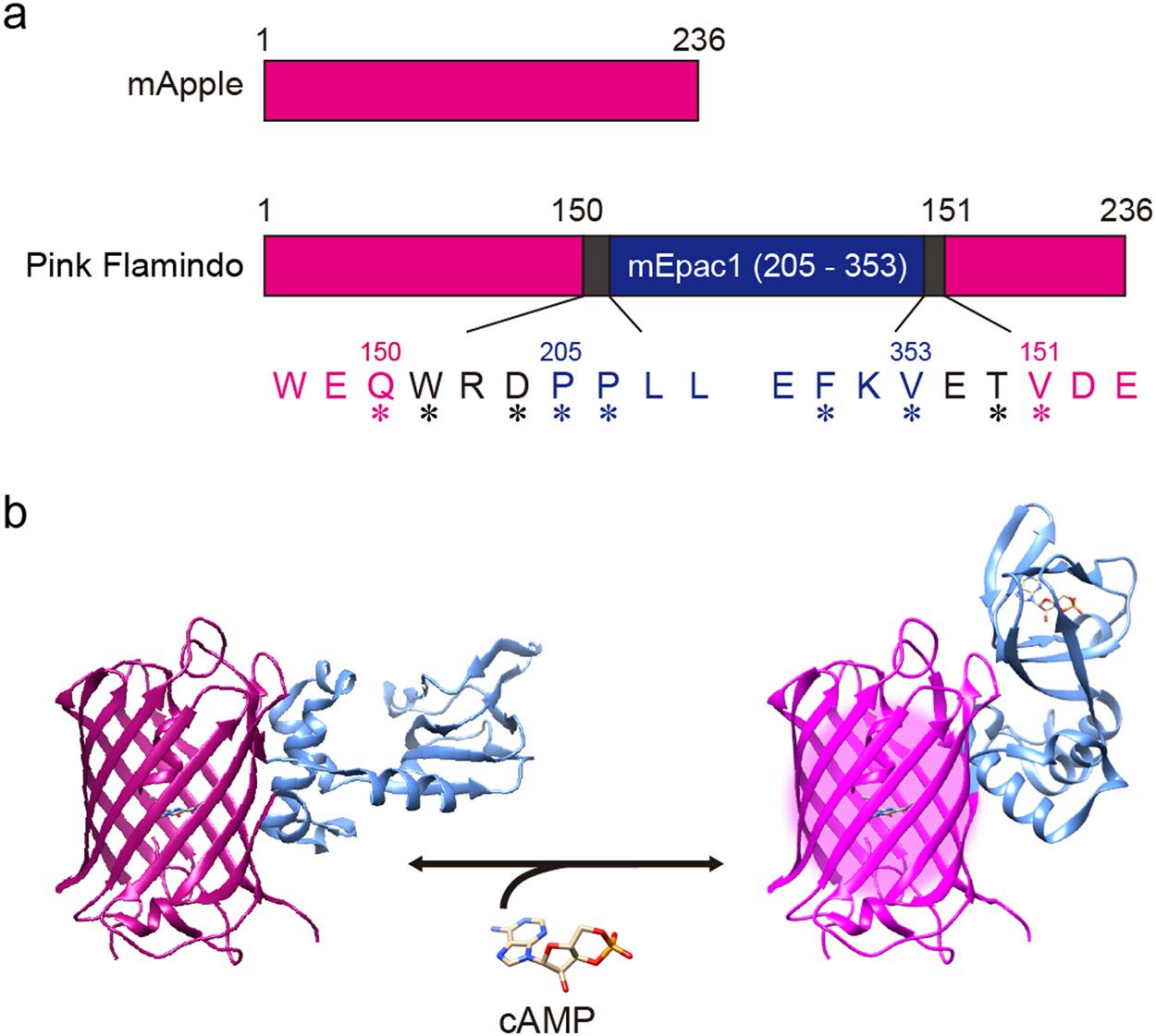
Schematic of the structure of Pink Flamindo. (**a**) Drawings for mApple and resultant Pink Flamindo. Asterisks indicate mutations. (**b**) 3D representation of Pink Flamindo bound (right) and unbound (left) to cAMP. Images were created using structural graphics from mCherry (PDB_4ZIO), which has the same origin of gene as mApple and Epac1 (cAMP-unbound: PDB_2BYV, cAMP-bound: PDB_4MGK).

Since cAMP functions in concert with various other intracellular signalling molecules, expansion of the colour palette of genetically-encoded FP-based cAMP indicators is a key requirement for studies aimed at delineating the interplay and/or hierarchy between cAMP and these molecules. The green FP-based cAMP indicator, Flamindo2, was originally constructed by fusing the green FP variant, Citrine, and the cAMP binding domain of the mouse exchange protein that is directly activated by cAMP 1 (Epac1, NP_ 01171281, 199–358 aa) to two linker peptides^9^. In this study, we inserted the same cAMP binding domain into a red FP variant, known as mApple, to create a red FP-based cAMP indicator^12^. We then produced numerous variants of the construct with different linker lengths and amino acid substitutions, in order to improve upon the dynamic range of the indicator. To achieve this aim, we followed the same method that we employed when previously developing a green cGMP indicator^13^. Interestingly, in the presence of cAMP, these variants showed increases or decreases of fluorescence intensity depending on the linker length (Supplementary Fig. S1). Of note, some variants with similar characteristics and structures of amino acids at the same residue showed similar responses (Supplementary Fig. S2). We found that one variant elicited a notable change in fluorescence intensity in response to cAMP (Fig. 1), and named this genetically-encoded red FP-based cAMP indicator “Pink Flamindo” (Pink Fluorescent cAMP indicator).

### Spectral properties of Pink Flamindo *in vitro*

**Figure 2:**
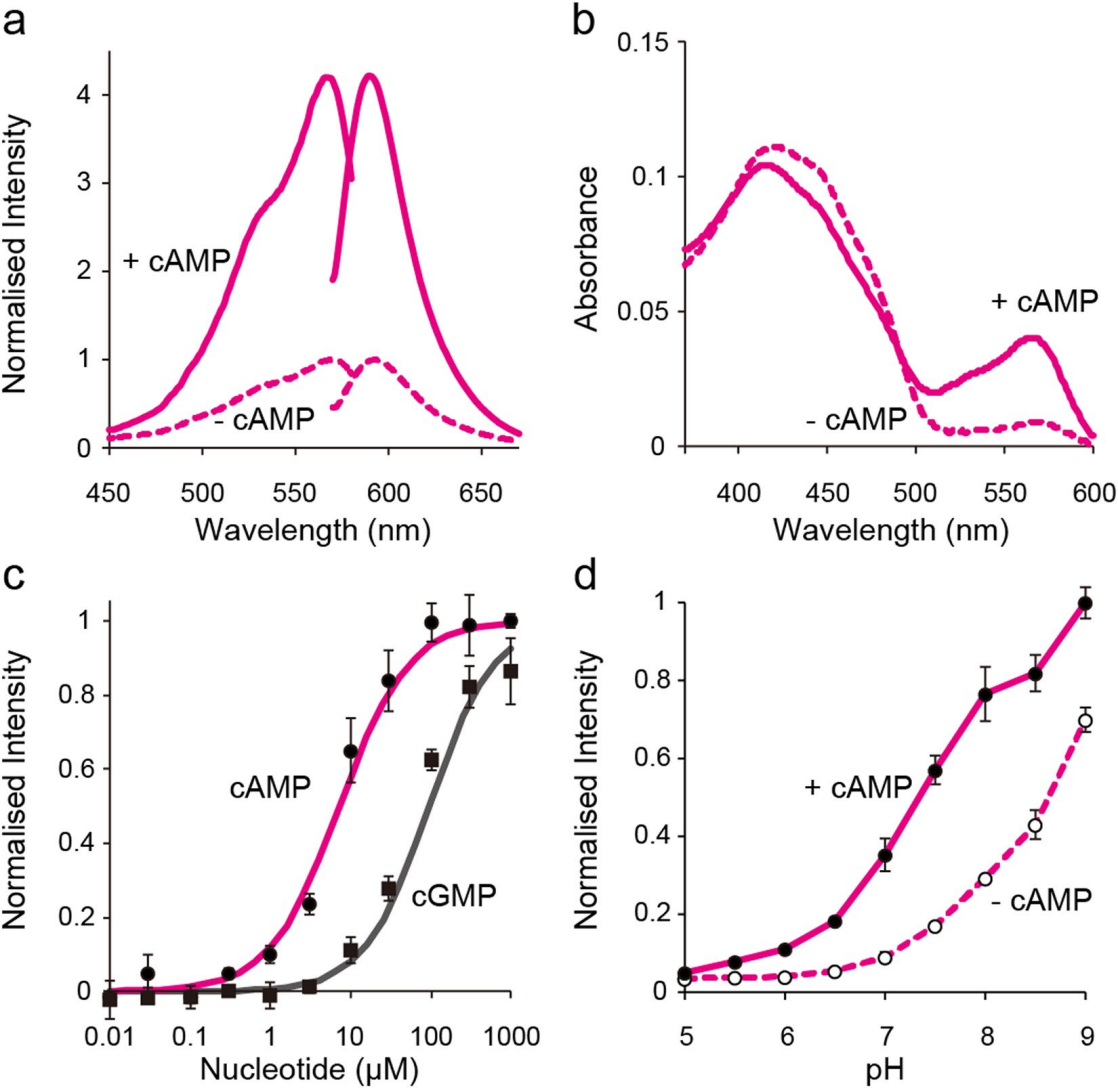
Spectroscopic characterisation of Pink Flamindo. (**a**) Excitation and emission spectra of purified Pink Flamindo protein in the presence (solid line) or absence (dashed line) of 100 μM cAMP. Each fluorescent intensity (FI) was normalised to the peak of FI in the absence of cAMP. (**b**) Absorbance spectra of 30 μM Pink Flamindo in the presence (solid line) or absence (dashed line) of 100 μM cAMP. (**c**) Dose-response curve of Pink Flamindo for cAMP (●, red line) and cGMP (■, gray line). *K*_d_ values calculated using the Hill equation were 7.2 μM for cAMP and 94 μM for cGMP. The peak of FI in the absence of cAMP or cGMP was normalised to 0, and the peak of FI in the presence of 1 mM cAMP was normalised to 1. The data represent the means ± standard deviation (n = 3). (**d**) Titration curves of Pink Flamindo against pH in the presence (●, solid line) or absence (○, dashed line) of 100 μM cAMP. The peak FI for each pH was normalised to the peak of FI in the presence of cAMP at pH 9.0. The data represent the means ± standard deviation (n = 3).

To investigate the properties of Pink Flamindo *in vitro*, we measured the fluorescence excitation and emission spectra of the purified Pink Flamindo protein. Pink Flamindo elicited an excitation peak at 567 nm and an emission peak at 590 nm (Fig. 2a). The fluorescence intensity of Pink Flamindo increased by 4.2-fold upon exposure to 100 μM cAMP, and the response was reversible (Supplementary Fig. S3a). Additionally, the fluorescence intensity of Pink Flamindo reached a peak within 5 sec even at low cAMP concentrations, which is comparable to the previous FRET-based indicator using Epac (Supplementary Fig. S3b)^5^. It is worth mentioning that the fluorescence intensity of Pink Flamindo is around 20-fold (without cAMP)/5-fold (with cAMP) lower than that of a red fluorescent protein derived from same origin, mCherry (Supplementary Fig. S4). To improve brightness, incubation of Pink Flamindo-expressing cells at lower temperature is required for accelerating chromophore maturation (Supplementary Fig. S5)^14^. The absorption spectra of 30 μM Pink Flamindo protein after the addition of cAMP decreased at 420 nm, and increased at 565 nm (Fig. 2b). mApple has an absorption peak at ~400 nm, which corresponds to the protonated form of the chromophore, and another peak at ~550 nm, which corresponds to the deprotonated form^15^. Given these data, it seems that Pink Flamindo changes the protonated/deprotonated ratio of its chromophore upon binding to cAMP, which leads to an increase in its fluorescence intensity.

We next examined the dose-response relationship of Pink Flamindo with cAMP or cGMP and calculated the dissociation constants (*K*_d_) (Fig. 2c). According to the Hill equation, the cAMP *K*_d_ = 7.2 μM and the cGMP *K*_d_ = 94 μM. Both these values are slightly higher than those of Flamindo2 (cAMP *K*_d_ = 3.2 μM and cGMP *K*_d_ = 22 μM)^9^. Flamindo2 was generated from Flamindo by modification of only the linker length. As previously discussed, we developed Pink Flamindo by also introducing several point mutations into mEpac1, the linker region and the FP sequence. These mutations drastically improved the dynamic range of the indicator. Although the specificity of Pink Flamindo to cAMP was retained, a subtle reduction in the affinity for cAMP was noted. Because previous studies have developed various FRET-based cAMP indicators with *K*_d_ between 50 nM and 10 μM^6, 16–19^ and successfully monitored physiological cAMP changes, Pink Flamindo would be applicable for live-cell imaging analysis. Meanwhile, previous cAMP indicators with different affinities have shown different kinetics^5^, and thus careful choice of cAMP indicator with optimal affinity and kinetics is essential to elucidate the phenomenon of interest. The Hill coefficients for cAMP and cGMP were 1.01 and 1.07, respectively, suggesting that Pink Flamindo does not exhibit allosteric effects. As has been reported for many other single, FP-based indicators^20–22^, Pink Flamindo showed an increase in its fluorescent intensity as a result of pH elevation (Fig. 2d). We would, therefore, encourage careful interpretation or pH correction of the data obtained from imaging experiments using Pink Flamindo.

### Utility of Pink Flamindo in live-cell imaging and optogenetic studies

**Figure 3:**
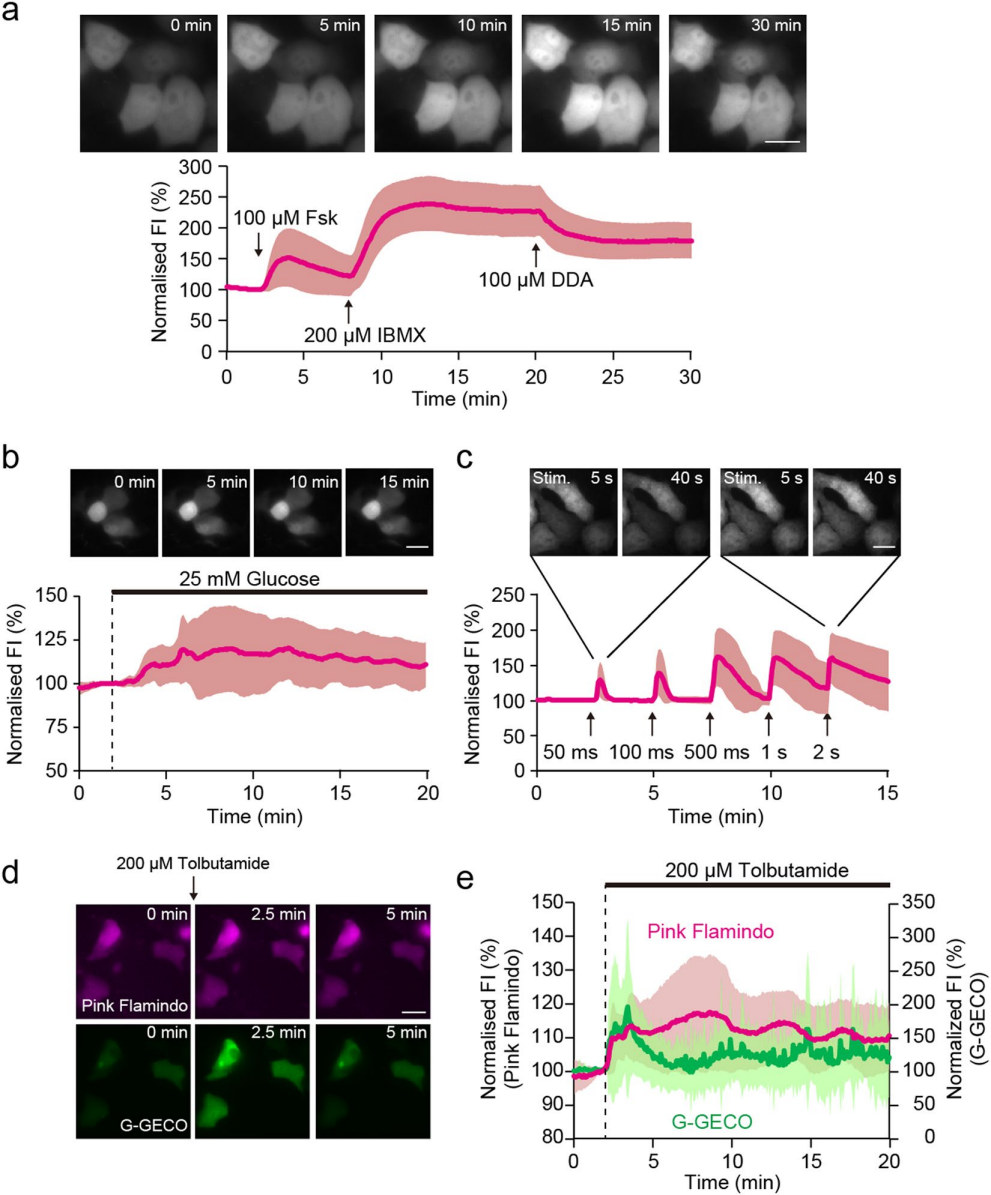
Live cell imaging using Pink Flamindo with photoactivated adenylyl cyclase, and dual-colour imaging with G-GECO. (**a**) Sequential images and time course of fluorescence intensity of Pink Flamindo expressed in HeLa cells after consecutive exposure to 100 μM Fsk, 200 μM IBMX and 100 μM DDA. Scale bar represents 20 μm. The data represent the means ± standard deviation (n = 30 cells from 5 experiments). (**b**) Sequential images and time course of fluorescence intensity of MIN6 m9 cells expressing Pink Flamindo during the application of 25 mM glucose. Scale bar represents 20 μm. The data represent the means ± standard deviation (n = 15 cells from 4 experiments). (**c**) Sequential images and time course of fluorescence intensity of HeLa cells co-overexpressing Pink Flamindo and photoactivated adenylyl cyclase (bPAC) upon blue light laser excitation at 1.8 μW. Scale bar represents 20 μm. The data represent the mean ± standard deviation (n = 23 cells from 4 experiments). (**d**) Sequential images of MIN6 m9 cells co-overexpressing Pink Flamindo and G-GECO after consecutive exposure to 200 μM tolbutamide. Scale bar represents 20 μm. (**e**) Time course of fluorescence intensity of MIN6 m9 cells co-overexpressing Pink Flamindo and G-GECO. The data represent means ± standard deviation (n = 18 cells from 4 experiments).

To investigate whether Pink Flamindo is functional for studies of living cells, we sequentially applied adenylyl cyclase activator, forskolin (Fsk), phosphodiesterase inhibitor, 3-isobutyl 1-methylxanthine (IBMX), and adenylyl cyclase inhibitor 2′,5′-dideoxyadenosine (DDA) to HeLa cells overexpressing Pink Flamindo. The fluorescence intensity of Pink Flamindo first showed a transient increase in the presence of 100 μM Fsk, followed by a long-lasting elevation in response to 200 μM IBMX, and finally a decrease in the presence of 100 μM DDA (Fig. 3a and Supplementary Movie S1). The application of DDA after exposure to Fsk and IBMX did not completely suppress the increase of fluorescence intensity. This would be ascribed to continuous suppression of phosphodiesterases by IBMX, because DDA only inhibits adenylyl cyclase. When stimulated with Fsk and IBMX, the fluorescence intensity of Pink Flamindo saturated at ~2.5-fold, which is lower than its dynamic range (4.2-fold) obtained from *in vitro* spectroscopic experiments (Fig. 1b). This difference may be explained by a certain basal level of cAMP in cells in a resting state^23^. Indeed application of 500 μM DDA to resting HeLa cells resulted in a decrease in the fluorescence intensity of Pink Flamindo (Supplementary Fig. S6a). As described before, purified Pink Flamindo protein has pH sensitivity in *in vitro* spectroscopic experiments (Fig. 2d). Therefore, we attempted to correct the effect of intracellular pH on the responses of Pink Flamindo by co-expressing the fluorescent pH indicator pHluorin in HeLa cells for future necessity. When we corrected responses of Pink Flamindo by 100 μM Fsk according to the previous study^21^ (also see Supplementary Methods), we confirmed that the intracellular pH changes can be cancelled by the correction (Supplementary Fig. S6b). Thus, we should critically evaluate the effect of pH sensitivity under situations when intracellular pH is likely to change. To study whether Pink Flamindo monitors cAMP elevation induced by physiological stimulation as well as pharmacological stimulation, we imaged mouse MIN6 m9 pancreatic β cells under glucose application^24^. Pancreatic β cells take up glucose and secrete insulin, which is partly mediated by the activation of adenylyl cyclases and cAMP production^25^. When we applied 25 mM glucose to MIN6 m9 cells, we found that the fluorescence intensity of Pink Flamindo showed a long-lasting elevation (Fig. 3b, Supplementary Movie S2). Taken together, these results suggest that Pink Flamindo is successfully used to monitor the intracellular dynamics of cAMP respond in response to both pharmacological and physiological stimulations in living cells.

We next examined whether Pink Flamindo can be used in conjunction with optogenetic tools — namely, photoactivated adenylyl cyclase (PAC), which artificially induces cAMP production following blue-light exposure^11^. When HeLa cells co-expressing Pink Flamindo and photoactivated adenylyl cyclase from *Beggiatoa sp*. (bPAC) were stimulated with 1.8 μW blue light laser (488 nm), we observed a rapid increase in fluorescence intensity. On the other hand, blue light laser stimulation itself induced little increase in the cells expressing Pink Flamindo only (Fig. 3c, Supplementary Fig. S7, Supplementary Movie S3). The peak amplitude and duration of responses were dependent on the duration of the blue light laser stimulation. These results suggest that we can control the amount and duration of intracellular cAMP production by bPAC activation. Therefore, we propose that monitoring the tightly controlled cAMP levels with Pink Flamindo or other red fluorescent probes is an appropriate approach to evaluate the physiological function of cAMP in living cells.

### Dual-colour imaging with Pink Flamindo and Ca^2+^ indicator, G-GECO

Ca^2+^ and cAMP are important signalling molecules that trigger hormone secretion in endocrine cells, including pancreatic β cells^25^. Here, we simultaneously monitored the dynamics of cAMP and Ca^2+^ in MIN6 m9 cells, by co-expressing Pink Flamindo and the green Ca^2+^ indicator G-GECO. Previous studies have shown that an increased level of ATP by glucose uptake closes ATP-sensitive K^+^ channels (K_ATP_ channels) in pancreatic β cells, which leads to Ca^2+^ influx and insulin secretion^26^. Here, application of 200 μM tolbutamide, which artificially closes K_ATP_ channels, induced an increase in the fluorescence intensity of both Pink Flamindo and G-GECO (Fig. 3d,e, Supplementary Movie S4). The fluorescence intensity of Pink Flamindo showed a single, sharp increase before gradually returning to baseline. Conversely, G-GECO exhibited long-lasting oscillations after reaching a peak, which is consistent with data from a previous observation^27^. We previously showed that extracellular Ca^2+^ influx activates Ca^2+^-sensitive adenylyl cyclase in MIN6 cells^8^, and these latest results further support this concept. These data suggest that Pink Flamindo is suitable for dual-colour imaging in combination with green fluorescent indicators.

### Application to *in vivo* two-photon imaging in cerebral cortical astrocytes

**Figure 4:**
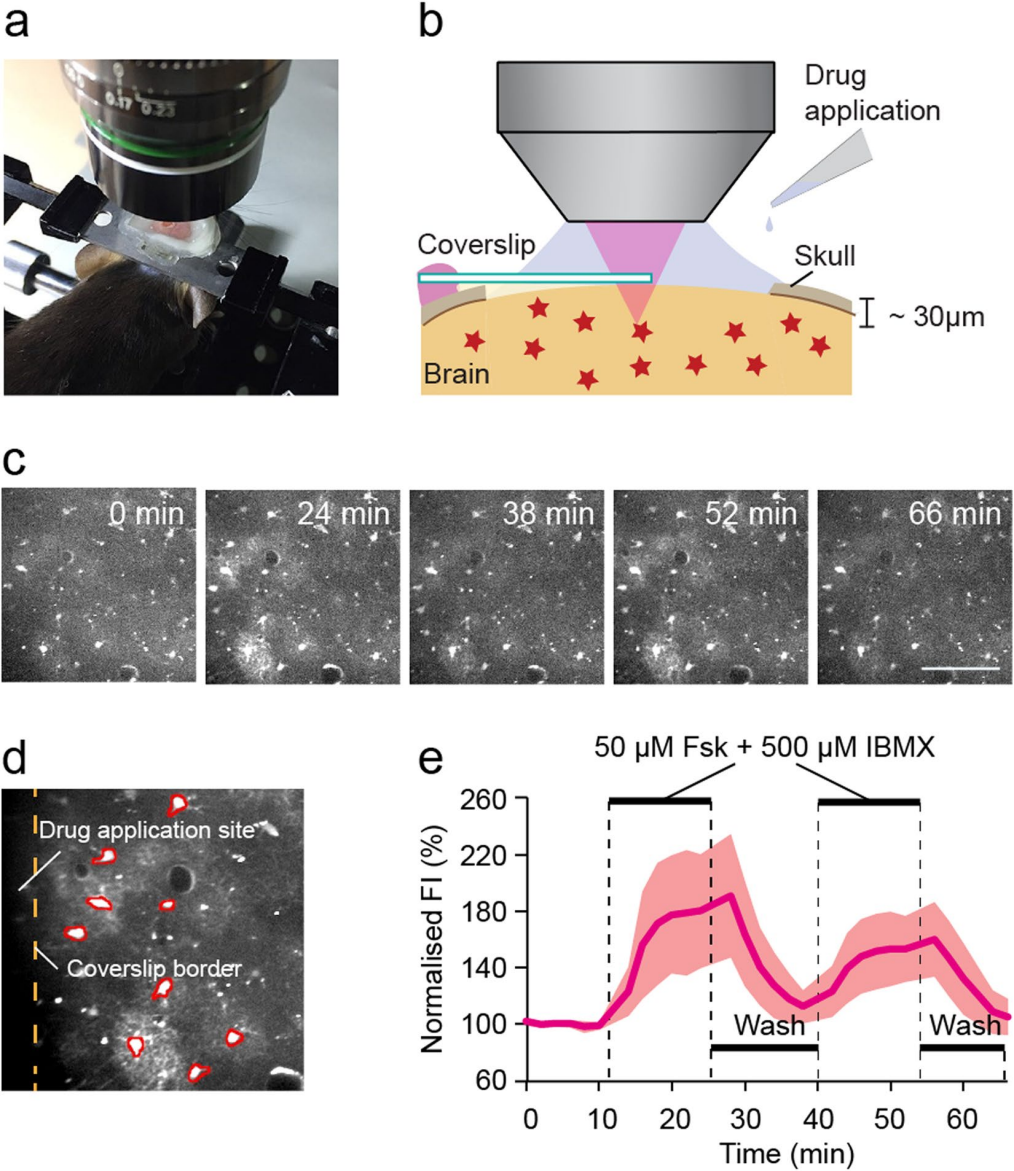
*In vivo* imaging of cerebral cortical astrocytes using Pink Flamindo. (**a**) Image showing the experimental setup of a mouse under anaesthesia, positioned under a two-photon microscope. (**b**) Schematic of the cranial window for *in vivo* two-photon imaging. (**c**) Sequential images of cortical astrocytes expressing Pink Flamindo during exposure to 50 μM Fsk and 500 μM IBMX. Scale bar represents 100 μm. (**d**) Enlarged image of **c** (24 min) to indicate the regions of interest. Analysed cells are marked in red. Yellow dashed line indicates the coverslip border (see **b**). Scale bar represents 50 μm. (**e**) Population mean trace of fluorescence intensity for Pink Flamindo from the images shown in **c**. The topical application of Fsk, IBMX and compound washout (indicated by black horizontal bars) was repeated twice. The data represent the means ± standard deviation (n = 9 cells).

We next investigated whether Pink Flamindo could be applied to *in vivo* brain imaging studies of mice. We used a recombinant adeno-associated virus with the human GFAP promoter to specifically express Pink Flamindo in cerebral cortical astrocytes (Supplementary Fig. S8a). Immunohistochemical analyses showed that the expression of Pink Flamindo was cytosolic and co-localised with the astrocyte maker S100B (Supplementary Fig. S8b). We also found that Pink Flamindo was expressed in the somata, fine processes, and endfeet of astrocytes. Individual Pink Flamindo-expressing astrocytes were visualised by two-photon microscopy through an acutely-prepared cranial window made through the skull of mice that were maintained under urethane anaesthesia (Fig. 4a,b). Topical application of 50 μM Fsk and 500 μM IBMX resulted in an increase of fluorescence intensity of Pink Flamindo in the imaged astrocytes (Fig. 4c,d and e, Supplementary Movie S5). The fluorescence intensity of Pink Flamindo returned to baseline following wash-out of both compounds. The elevation of fluorescence intensity for Pink Flamindo could be repeated with a second application of Fsk and IBMX. The population mean increase in fluorescence intensity exhibited by the astrocyte somata, 10 minutes after the first exposure to Fsk and IBMX, was 55.5 ± 13.3% (n = 5 mice; data represent the means ± SEM). The analysis for the Flamindo2, turn-off type green fluorescent indicator, also showed recovery of fluorescence intensity after wash-out of the compounds, and yielded a signal decrease of 64.5 ± 10.9% (n = 5 mice; data represent the means ± SEM) (Supplementary Fig. S8c,d). The opposing responses of Pink Flamindo and Flamindo2 support the proper response of these cAMP indicators *in vivo*. The magnitude of the change in fluorescence was comparable between Pink Flamindo and Flamindo2, with a similar dynamic range (4.2 and 4.0 fold, respectively, *p* = 0.62) and dissociation constant (7.2 μM and 3.2 μM, respectively). These data are consistent with our previous spectroscopic results obtained *in vitro*.

Astrocytes are the most abundant glial cells in the cerebral cortex and they express various G protein-coupled receptors (GPCRs) for neuromodulators, such as acetylcholine and noradrenaline. These neuromodulators are associated with brain states, such as vigilance and startle, and influence wide brain areas by volume transmission and activating astrocytic GPCRs^28^. Given these data, we propose that Pink Flamindo will serve as a potent tool to investigate physiological Gs-GPCR or Gi-GPCR activation in astrocytes and other brain cells, especially in combination with multi-colour imaging and optogenetics.

## Conclusion

This report describes the generation of the first red FP-based cAMP indicator, named Pink Flamindo. Spectroscopic analyses showed that the fluorescence intensity of Pink Flamindo increases with the cAMP concentration in a dose-response manner. The development of red FP cAMP indicator allowed dual cAMP/Ca^2+^ imaging as well as cAMP imaging in conjunction with optogenetic stimulation by blue light. These results demonstrate that Pink Flamindo overcomes the limited utility of FRET-based or green FP-based indicators due to the spectral overlap with other fluorescence indicators or optogenetic molecules. Moreover, we were able to apply Pink Flamindo to *in vivo* two-photon imaging of cerebral cortical astrocytes. This application expands the utility of this indicator to elucidate the complex interplay and hierarchy between different signalling molecules at a high spatiotemporal resolution in living animals. The use of our novel indicator in optogenetic studies and *in vivo* imaging will open new avenues for investigation of the complex signalling networks between various molecules in cells, tissues, organs and whole animals.

## Methods

### Chemicals

cAMP and 2′,5′-dideoxyadenosine (DDA) were purchased from Sigma-Aldrich (St. Louis, MO, USA). Forskolin (Fsk) and nigericin were purchased from WAKO Pure Chemical Industries (Osaka, Japan). cGMP and 3-isobutyl 1-methylxanthine (IBMX) were purchased from Merck Millipore (Darmstadt, Germany).

### Plasmid construction

The DNA fragment of a red fluorescent protein variant, mApple, utilized in a red Ca^2+^ indicator R-GECO^12^ was created by DNA synthesis from Integrated DNA Technologies (Coralville, IA, USA). It was engineered to contain *Sac*II and *Eco*RI restriction enzyme sites between A150 and V151 and cloned into the pRSET-A vector. The cAMP binding domain of mEpac1 (NM_001171281, 199–358 amino acids) derived from Flamindo2 and was inserted into the *Sac*II/*Eco*RI sites of mApple. To improve the performance of the indicator, a number of variants were created with the addition or deletion of linker amino acids in the both N-terminus and C-terminus of the cAMP binding domain by PCR. Random, site-directed mutations were also introduced into the construct by PCR with sense and anti-sense primers including NNK and MNN, respectively. The mutant construct that elicited the highest dynamic range was selected and named Pink Flamindo. Pink Flamindo was cloned into the pcDNA3.1 (-) vector for expression in mammalian cells. The humanised photoactivated adenylyl cyclase from *Beggiatoa sp*. (h_bPAC) in a pGEM-HE vector was obtained from Addgene (#28134, Cambridge, MA, USA), and was subcloned into the *Bgl*II/*Eco*RI sites of the pEGFP-C1 vector. The G-GECO plasmid was also obtained from Addgene (#32447). The Flamindo2 plasmid was developed in our previous study^9^.

### Protein expression and *in vitro* spectroscopy

For protein expression, Pink Flamindo or mCherry in the pRSET-A vector was transformed into *Escherichia coli* JM109 (DE3) cells, and the cells were cultured for 4 days at 20 °C and harvested by centrifugation. The harvested cells were suspended in phosphate-buffered saline (PBS) and lysed by three freeze-thaw cycles and sonication with 40 μg/mL lysozyme. After centrifugation, the supernatants containing the Pink Flamindo protein were collected and purified on an Ni-NTA agarose column (QIAGEN, KJ Venlo, Netherlands) followed by clean up through a PD-10 gel filtration column (GE Healthcare) to remove imidazole and elution in HEPES buffer (150 mM KCl and 50 mM HEPES-KOH [pH 7.4]). To generate pH titration curves, purified Pink Flamindo protein was diluted in HEPES buffer (100 mM HEPES-KOH [pH 5 to 9]). The absorption spectra of purified Pink Flamindo protein were measured using an UV spectrophotometer (UV-1800, Shimadzu, Kyoto, Japan), and the fluorescence spectra were measured using a fluorescence spectrophotometer (F-2500, Hitachi, Tokyo, Japan).

### Cell culture and transfection

HeLa and MIN6 m9 cells were cultured in high glucose Dulbecco’s modified Eagle’s medium (Sigma-Aldrich) supplemented with 4.5 g/L glucose, L-glutamine, sodium pyruvate, 10% (v/v) heat-inactivated foetal bovine serum (Sigma-Aldrich), 100 U/mL penicillin and 100 μg/mL streptomycin (Sigma-Aldrich), at 37 °C in an atmosphere of 5% CO_2_. The media to culture MIN6 m9 cells also contained 50 μM 2-mercaptoethanol^24^. For imaging experiments, the cells were dissociated with trypsin and then plated onto glass coverslips coated with poly-L-lysine (Sigma-Aldrich) placed within 35-mm dishes. The cells were transfected with plasmids using Polyethyleneimine “MAX” (Polysciences, Warrington, PA, USA) or Lipofectamine 2000 Transfection Reagent (Thermo Fisher Scientific, Waltham, MA, USA), 2 days after plating. The media was exchanged 4 h after transfection and the cells were cultured at 32 °C for 2or 3 days until imaging.

### Visualisation of intracellular cAMP dynamics

HeLa and MIN6 m9 cells cultured in 35-mm dishes were washed twice and imaged in modified Ringer’s buffer (RB: 140 mM NaCl, 3.5 mM KCl, 0.5 mM NaH_2_PO_4_, 0.5 mM MgSO_4_, 1.5 mM CaCl_2_, 10 mM HEPES, 2 mM NaHCO_3_, 5 mM glucose for HeLa and 3 mM glucose for MIN6 m9 cells, respectively). MIN6 m9 cells were cultured in the medium containing 1 g/L glucose for overnight, and incubated in RB for 30 min at 37 °C before imaging. Dishes were mounted on a stage that was heated and maintained at 37 °C and imaging was performed using an inverted microscope (IX-71, Olympus, Tokyo, Japan) equipped with an oil-immersion objective lens (UApo/340, 40×, NA = 1.35, Olympus), an intermediate magnification lens (1.6×), and an EM-CCD camera (Evolve, Photometrics, Tucson, AZ, USA). Images were acquired using a xenon lamp, 545–580 nm excitation filter, 585 nm dichroic mirror and 610 nm emission filter (Olympus). The exposure time of the EM-CCD camera was controlled by MetaMorph software (Molecular Devices, Sunnyvale, CA, USA). Images were acquired every 5 sec for 30 min. Data analysis of the acquired images was performed using MetaMorph software and the fluorescence intensity of the cells was quantified. After subtracting the background, basal fluorescence intensity, normalised to 100%, was calculated as the average fluorescence intensity between 90 and 120 sec after the beginning of image acquisition.

### Imaging with photoactivated adenylyl cyclase stimulation and dual-colour imaging

HeLa and MIN6 m9 cells were washed and imaged as described above. Imaging was performed using an inverted microscope (ECLIPSE Ti-E, Nikon, Tokyo, Japan) equipped with an oil-immersion objective lens (CFI Plan Fluor, 40×, NA = 1.30 and an intermediate magnification lens (1.5×), Nikon) and an EM-CCD camera (iXon, Andor, Belfast, UK). For HeLa cells, images were acquired using an optically pumped semiconductor 488-nm laser (Sapphire 488LP, 30 mW, Coherent, Santa Clara, Canada) and a diode-pumped solid-state 561-nm laser (85-YCA-010, 10 mW, Melles Griot, Tokyo, Japan). For MIN6 m9 cells, images were acquired using a mercury lamp (Nikon). Two filter sets (465–495 nm excitation filter, 505 nm dichroic mirror, and 515–555 nm emission filter (Nikon) for G-GECO, and 540–580 nm excitation filter, 595 nm dichroic mirror and 600–660 nm emission filter (Nikon) for Pink Flamindo, respectively) were used and automatically exchanged by a filter turret (TI-FLC, Nikon). Image acquisition and quantification were performed using MetaMorph software as described above.

### Surgical procedures for virus inoculation

The procedures involving animal care, surgery and sample preparation were approved by the Animal Experimental Committee of RIKEN Brain Science Institute and performed in accordance with the guidelines of the Animal Experimental Committee of RIKEN Brain Science Institute (H27–2–230). The adeno-associated virus (AAV) pAAV-hGFAP-Pink Flamindo and pAAV-hGFAP-Flamindo2 constructs were synthesised by replacing the promoter and coding region of the pAAV-hSyn-EGFP vector (Addgene plasmid #50465, a gift from Bryan Roth) with the human GFAP promoter^29^ and Pink Flamindo or Flamindo2 sequence; the latter was inserted between the *Age*I and *Not*I sites. AAV9-hGFAP-Pink Flamindo and AAV-hGFAP-Flamindo2 was purified at a titer of 6.6 × 10^13^ vg/mL and 1.1 × 10^14^ vg/mL, respectively. The AAV was diluted with PBS for microinjection, to 6.6 × 10^12^ vg/mL and 1.1 × 10^13^ vg/mL for Pink Flamindo and Flamindo2, respectively. For microinjection, mice were initially anesthetised by intraperitoneal injection containing a ketamine cocktail (ketamine 70 mg/kg, xylazine 10 mg/kg), and then fixed in a stereotaxic frame. The mice were then maintained under an anaesthetic of 1% isoflurane for the duration of the experiment. A small craniotomy was made and a glass micropipette containing AAV was inserted to the primary somatosensory cortex at a 20° angle. Microinjection of 500 nL AAV was made over 5 min using a FemtoJet injector (Eppendorf, Hamburg, Germany) at depths of 350 μm and 500 μm below the surface. *In vivo* imaging experiments were performed 2 weeks after viral inoculation. Immunohistochemistry for Pink Flamindo and S100B was performed using anti-DsRed (1:1000, Clontech #632496, Shiga, Japan) and anti-S100B (1:1000, #S2532, Sigma-Aldrich) antibodies.

### Cranial window preparation and *in vivo* two-photon imaging

On the day of imaging, mice were anesthetised with urethane by intraperitoneal injection (1.5 g/kg) and the body temperature was maintained at 37 °C with a heating pad (BWT-100A, Bio Research Centre, Nagoya, Japan, or TR-200, Fine Science Tools, Foster City, CA, USA) during the course of the surgery and recordings. The skull was exposed by making an incision to the scalp, and a metal frame was then attached to the skull using a dental acrylic (Fuji LUTE BC, GC Corporation, Tokyo, Japan, and Super Bond C&B, Sun Medical, Shiga, Japan). A craniotomy (3 mm in diameter) was made above the virus injection site and the dura mater was surgically removed. After filling the craniotomy with artificial cerebrospinal fluid (aCSF), ~50% of the craniotomy area was gently sealed with a thin glass coverslip (3 mm × 3 mm, thickness: 0.12 mm, Matsunami Glass, Osaka, Japan), leaving an open area for drug application. The cranial window was fixed with dental cement.

*In vivo* imaging was performed using a resonant scanner-based two-photon microscope (B-Scope, Thorlabs, Newton, NJ, USA) with a Chameleon Ultra 2 laser (Coherent, wavelength 1040 nm and 960 nm for Pink Flamindo and Flamindo2, respectively) and an objective lens (XLPlan N 25×, NA = 1.05, Olympus). The laser power under the objective lens was ~30 mW. The B-Scope was equipped with a reverse dichroic mirror (ZT405/488/561/680-1100rpc, Chroma, Bellows Falls, VT, USA) and the emission light was separated using a dichroic mirror (FF562-Di03, Semrock, Rochester, NY, USA) with bandpass filters FF03-525/50 and FF01-607/70 (both from Semrock) for the green and red channels, respectively. Twenty consecutive images were acquired at a frame rate of 1 frame/sec, where each image was an internally averaged image of 30 frames within a second. Image acquisition was performed every 2 min using ThorImage software (Thorlabs) and the 20 images were averaged to represent the time point.

For data analysis, an offset value, calculated as the mean of 100 lowest pixel values, was subtracted from each frame. Astrocyte somata were marked manually using ImageJ software (National Institutes of Health, Bethesda, MD, USA), and the relative intensity change (ΔF/F, fold change) was calculated from (F_max_− F_0_)/F_0_ for Pink Flamindo, and from (F_0_ − F_min_)/F_min_ for Flamindo2.

### Statistical Analysis

The data shown represent the means ± standard deviation (SD) or standard error of the mean (SEM). Means were compared by two-sided Welch’s *t* test or one-way ANOVA with Tukey’s multiple comparisons.

## Electronic supplementary material

Movie S1

Movie S2

Movie S3

Movie S4

Movie S5

Supplementary Information
