# Supplementary material for: Red fluorescent protein-based cAMP indicator applicable to optogenetics and in vivo imaging
Source: Sci Rep. 2017 Aug 4;7:7351. doi: 10.1038/s41598-017-07820-6 (PMC5544736; doi:10.1038/s41598-017-07820-6)
Supplement: Supplementary file 6 — Supplementary Information [file 41598_2017_7820_MOESM6_ESM.doc]

**Online Supplementary Information**

**Red fluorescent protein-based cAMP indicator applicable to optogenetics and *in vivo* imaging**

Kazuki Harada^1, a^, Motoki Ito^2, a^, Xiaowen Wang^3^, Mika Tanaka^3^, Devina Wongso^4^, Ayumu Konno^5^, Hirokazu Hirai^5^, Hajime Hirase^3^, Takashi Tsuboi^1, 2, *^ and Tetsuya Kitaguchi^4, 6, *^

^1^ Department of Life Sciences, Graduate School of Arts and Sciences, The University of Tokyo, 3-8-1 Komaba, Meguro, Tokyo 153-8902, Japan

^2^ Department of Biological Sciences, Graduate School of Science, The University of Tokyo, 7-3-1 Hongo, Bunkyo, Tokyo 113-0033, Japan

^3^ Laboratory for Neuron-Glia Circuitry, RIKEN Brain Science Institute, Hirosawa 2-1, Wako-shi, Saitama 351-0198, Japan

^4^ Cell Signaling Group, Waseda Bioscience Research Institute in Singapore (WABIOS), 11 Biopolis Way, #05-02 Helios, Singapore 138667, Singapore

^5^ Department of Neurophysiology and Neural Repair, Gunma University Graduate School of Medicine, Maebashi, Gunma 371-8511, Japan

^6^ Comprehensive Research Organization, Waseda University, #304, Block 120-4, 513 Wasedatsurumaki-cho, Shinjuku, Tokyo 162-0041, Japan

^*^ Tetsuya Kitaguchi, Ph.D. E-mail: kitaguct-gfp@umin.ac.jp

^*^ Takashi Tsuboi, Ph.D. E-mail: takatsuboi@bio.c.u-tokyo.ac.jp

^a^ These authors contributed equally.


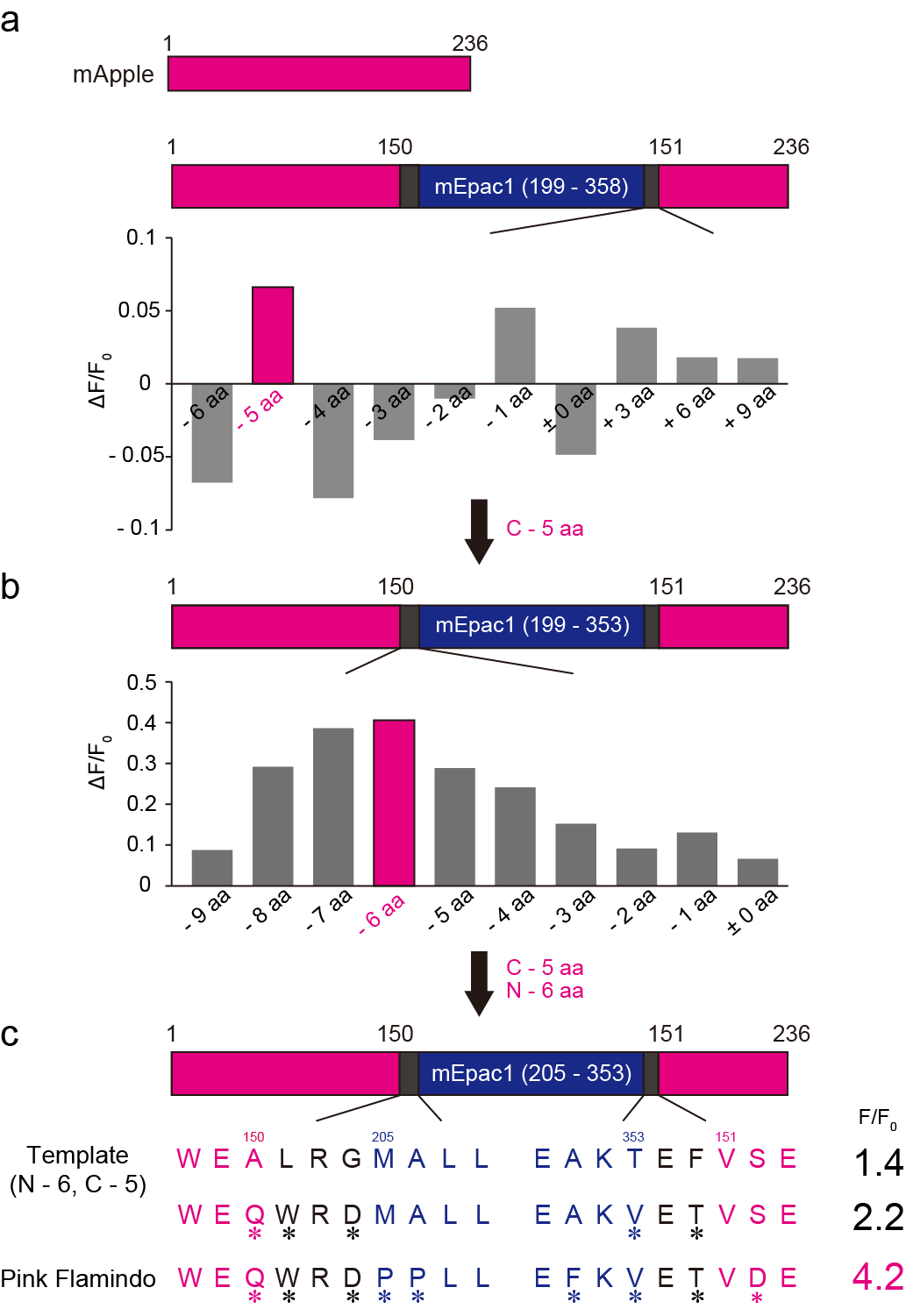
**Supplementary Figure S1.** **Generation and screening process of the Pink Flamindo construct.** (**a, b**) Schematic of mApple and the indicator prototype, and the screening results of various candidate constructs with a modified C-terminus (**a**) and N-terminus (**b**) linker length. Responses to 100 μM cAMP were compared. (**c**) Location of point mutations in the candidate construct with an optimal linker length (N minus 6 amino acids, C minus 5 amino acids). Asterisks represent the introduced mutations. The dynamic range (F/F_0_) of each construct in response to 100 μM cAMP was compared and the construct with the highest F/F_0_ was selected to carry forward and named Pink Flamindo.


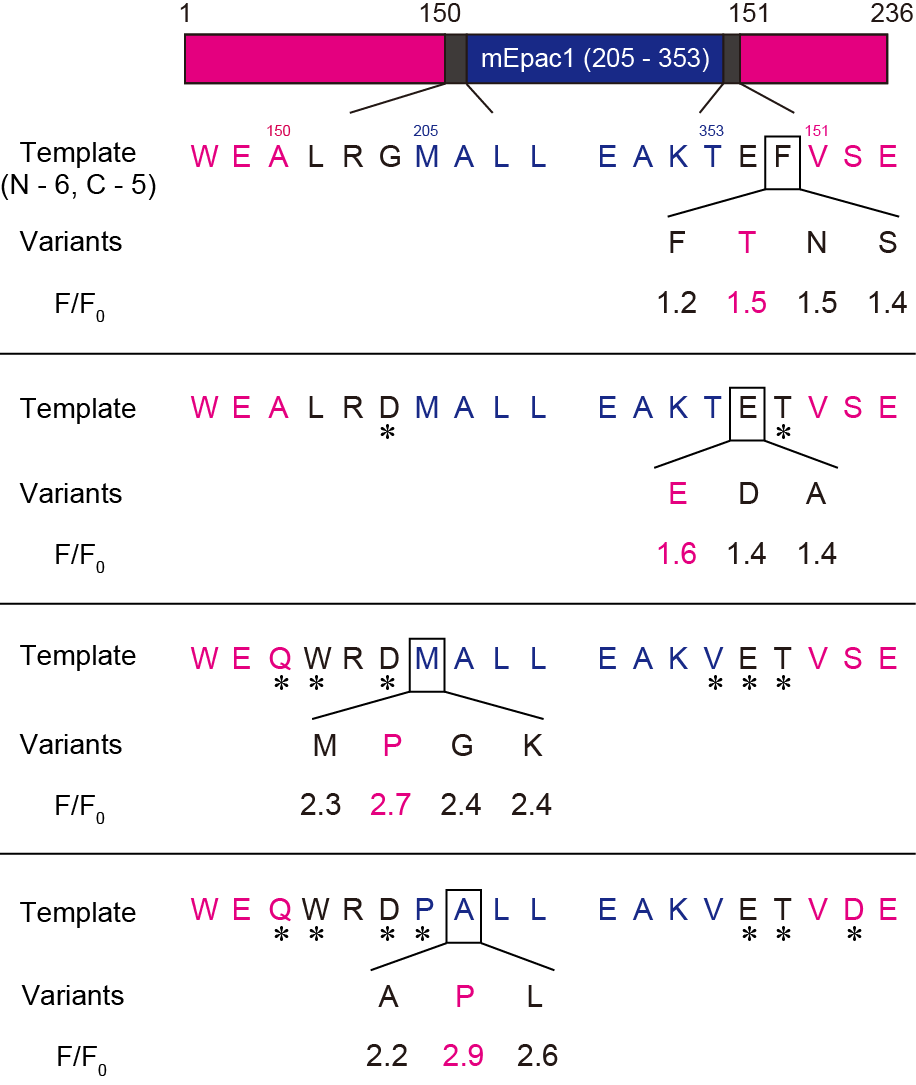
**Supplementary Figure S2.** **Typical results of introduced random point mutations during the screening process of Pink Flamindo.** Variants with the two or three highest F/F_0_ for each amino acid residue are shown.


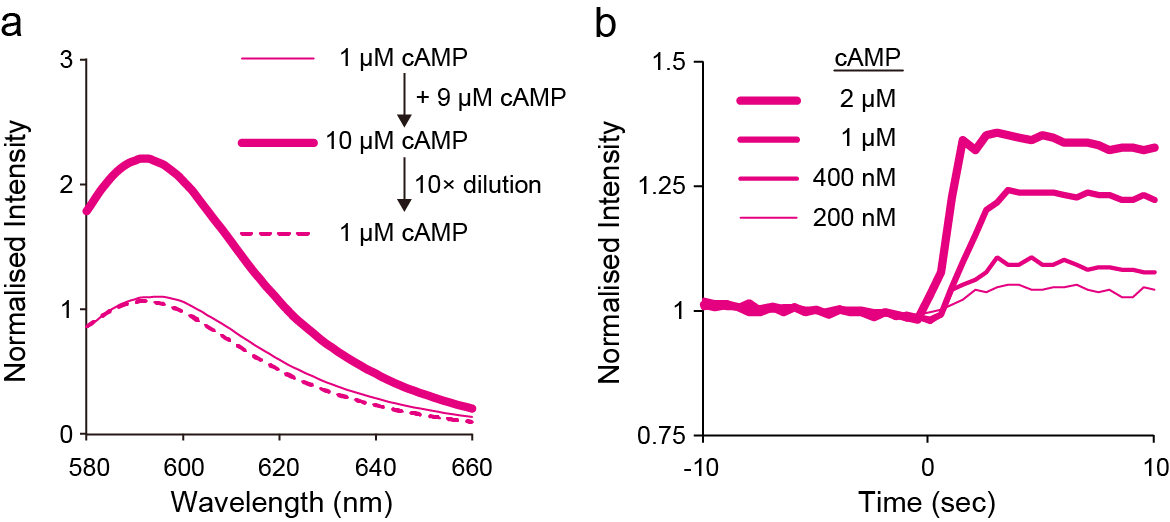
**Supplementary Figure S3.** **Reversibility and kinetics in the responses of Pink Flamindo.**

(**a**) Emission spectra of purified Pink Flamindo protein in the presence of 1 μM cAMP (solid, light magenta line), 10 μM (solid, magenta line), and 1 μM cAMP after dilution of 10 μM cAMP (dashed line). The peak of fluorescence intensity (FI) in the absence of cAMP was normalised to 1. (**b**) Time courses of FI for Pink Flamindo upon application of various concentrations of cAMP.


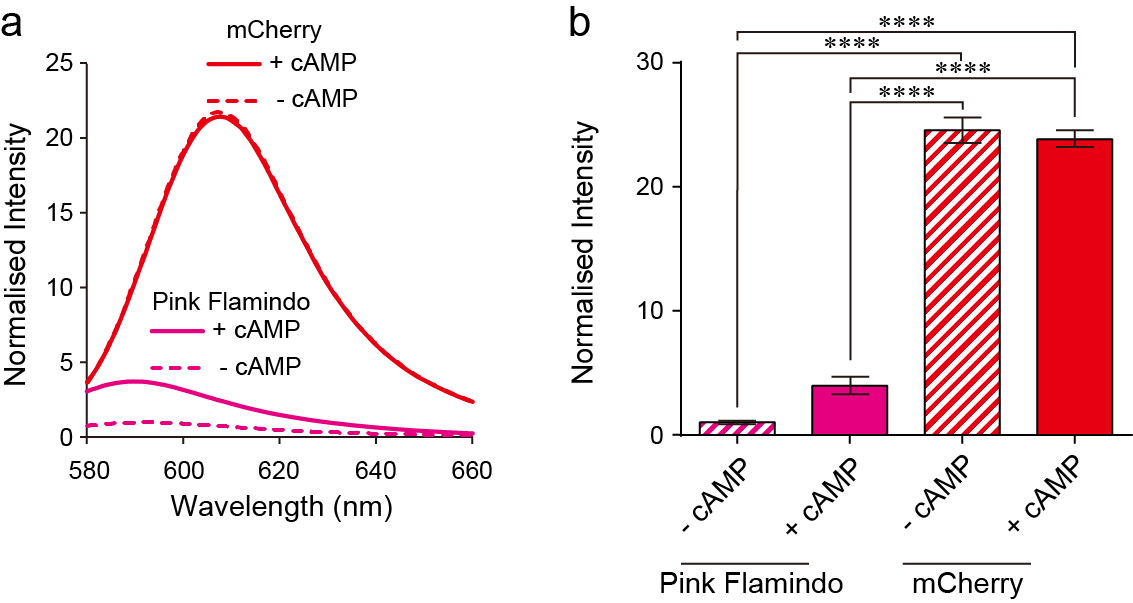
**Supplementary Figure S4. Basal brightness of purified Pink Flamindo protein.** (**a**) Emission spectra of purified Pink Flamindo (magenta lines) and mCherry (red lines) proteins in the presence (solid lines) and absence (dashed lines) of 100 μM cAMP. The peak of fluorescence intensity (FI) in Pink Flamindo in the absence of cAMP was normalised to 1. (**b**) Comparison of FI from the emission spectra shown in **a**. n =3 experiments. The data represent the means ± standard error of the mean. ****, *p* < 0.0001.


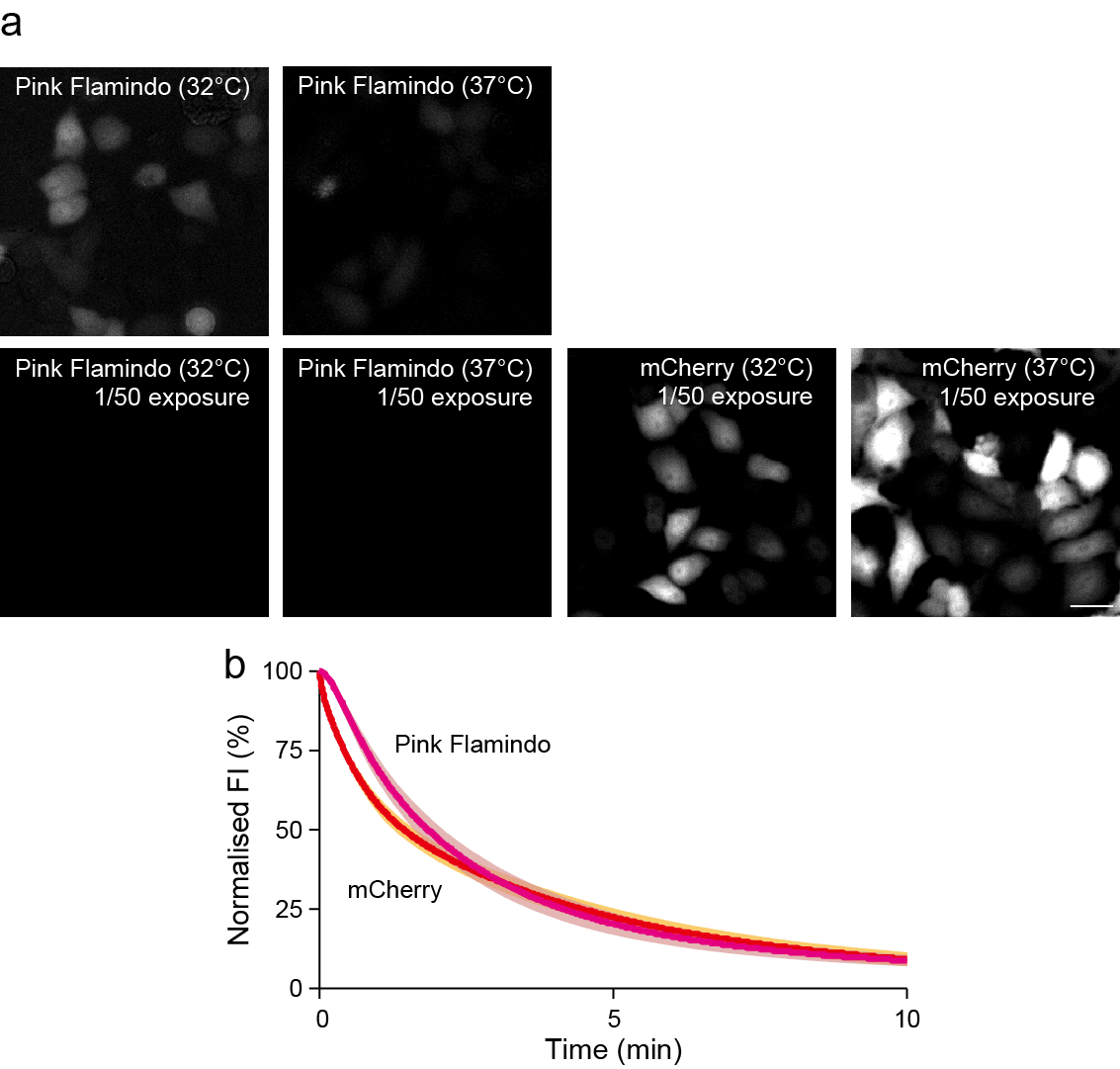
**Supplementary Figure S5. Basal brightness and photobleach kinetics of Pink Flamindo in living cells.** (**a**) Basal brightness of Pink Flamindo and mCherry expressed in HeLa cells (32°C or 37°C) at the exposure of 500 msec (top) and 10 msec (bottom). Note that Pink Flamindo shows higher fluorescence intensity at 32°C while mCherry shows higher at 37°C. Scale bar represents 30 μm. (**b**) Photobleach curves of Pink Flamindo and mCherry expressed in HeLa cells at 32°C under exposure to 2.6 W/cm^2^ of green light. Note that Pink Flamindo and mCherry shows comparable kinetics. The data represent the means ± standard deviation (n ≥ 7 cells from 3 experiments).


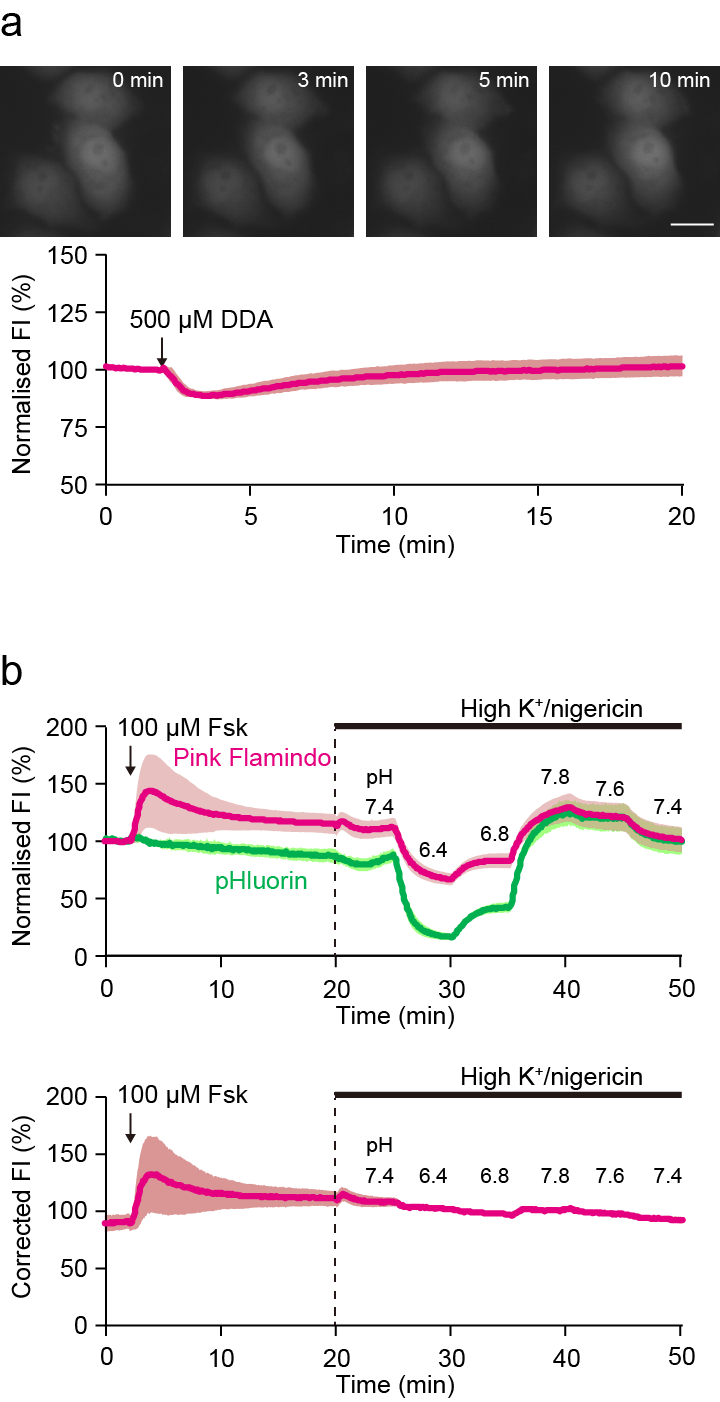
**Supplementary Figure S6. Investigation of basal cAMP production and correction of intracellular pH effects on the response of Pink Flamindo.** (**a**) Sequential images and time course of fluorescence intensity of Pink Flamindo expressed in resting HeLa cells following application of 500 μM DDA. Scale bar represents 20 μm. The data represent the means ± standard deviation (n = 17 cells from 3 experiments). (**b**) (Top) Time course of fluorescence intensity of HeLa cells co-expressing Pink Flamindo (magenta line) and pHluorin (green line) following application of 100 μM Fsk, and perfusion with high K^+^/nigericin solution. at pH 6.4-7.8. (Bottom) Time couse of fluorescence intensity in Pink Flamindo after correction of pH effects. The data represent the means ± standard deviation (n = 16 cells from 3 experiments).


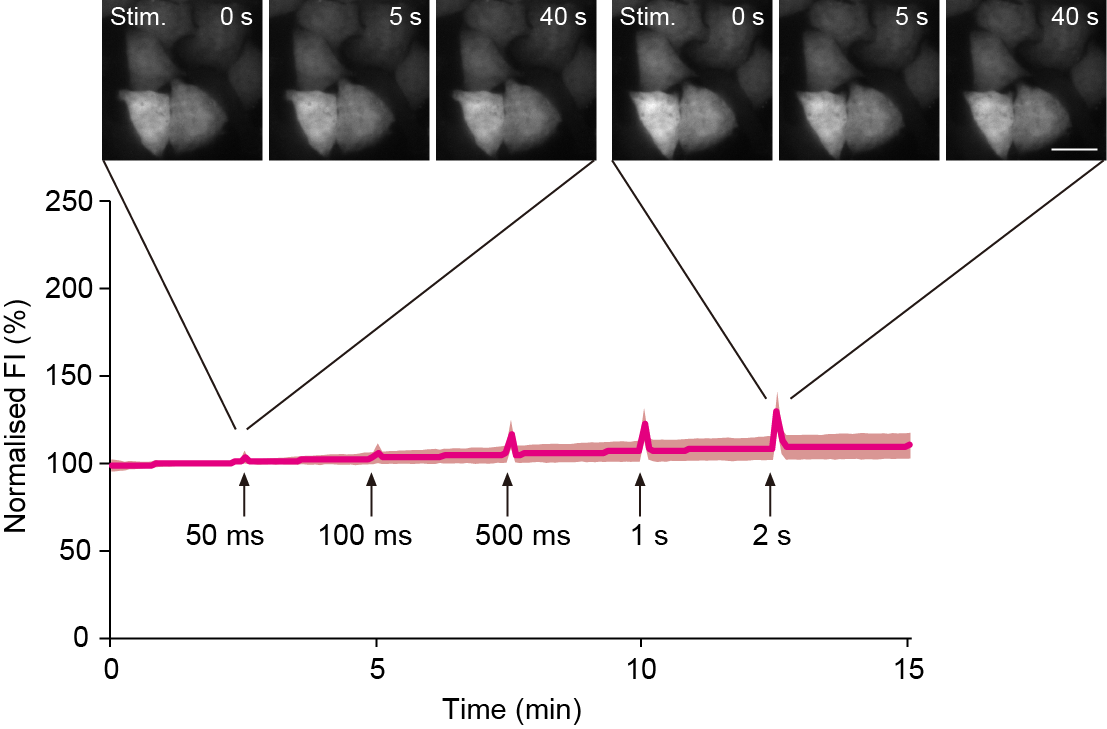
**Supplementary Figure S7.** **Changes in fluorescence intensity of Pink Flamindo exposed to blue light laser in the cells expressing Pink Flamindo only.** Sequential images and time course of fluorescence intensity of HeLa cells expressing only Pink Flamindo upon blue light excitation at 1.8 μW. Scale bar represents 20 μm. The data represent the means ± standard deviation (n = 14 cells from 3 experiments).


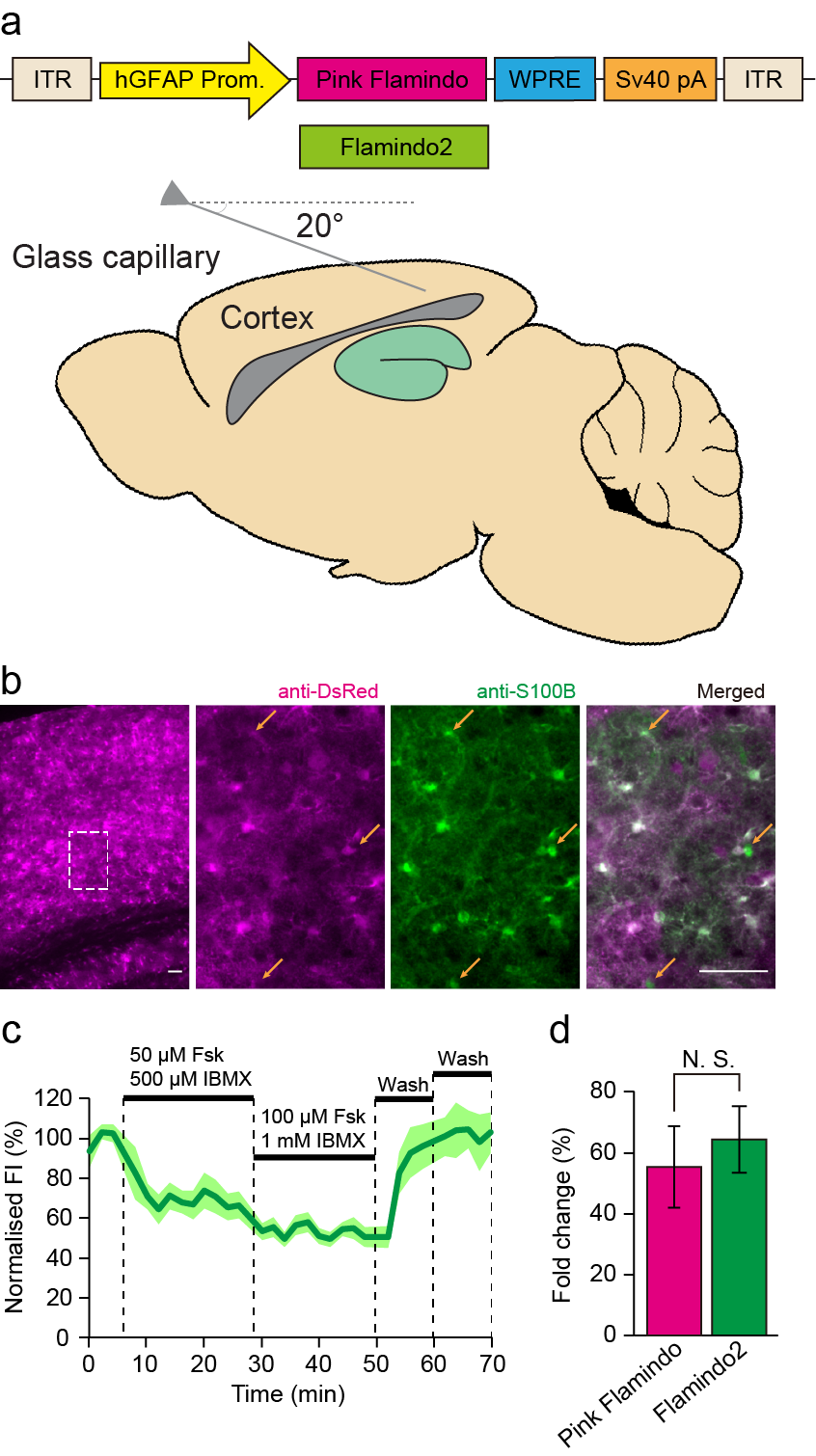
**Supplementary Figure S8. Virus inoculation of Pink Flamindo or Flamindo2 into cerebral cortical astrocytes, and *in vivo* imaging of Flamindo2.** (**a**) Recombinant adeno-associated virus vector design for pAAV-GFAP-Pink Flamindo or pAAV-GFAP-Flamindo2 and schematic of the cerebral viral delivery procedure. (**b**) Immunohistochemical analysis of Pink Flamindo expression in the somatosensory cortex, 1 month after viral inoculation. The dotted square was magnified in the following three panels. Most Pink Flamindo-expressing cells (DsRed immunofluorescence, magenta) are positive for S100B expression (green), as visualized in white in the merged image. A few cells in the images express S100B but not Pink Flamindo (arrows). Scale bars represent 100 μm. (**c**) Population mean trace of fluorescence intensity of Flamindo2 during a 70-minute session of exposure to 50 μM forskolin (Fsk), 500 μM 3-isobutyl-1-methylxanthine (IBMX), 100 μM Fsk, 1 mM IBMX, and compound washout. The data represent the means ± standard deviation (n = 7 cells). (**d**) Statistics for population mean of the Pink Flamindo (n = 5 mice) and Flamindo2 (n = 5 mice) signal increases for individual animals. The data represent the means ± standard error of the mean. Abbreviations: ITR, inverted terminal repeats; N. S., not significant; WPRE, woodchuck hepatitis virus post-transcriptional regulatory element.

**Supplementary Methods**

**Investigation of reversibility to cAMP in the responses of Pink Flamindo *in vitro***

Emission spectra of purified Pink Flamindo protein were measured in the absence and presence of 1 or 10 μM cAMP as described in the main text. Subsequently, samples with 10 μM cAMP were diluted to 10-fold in (A) PBS or (B) PBS with 10 μM cAMP. After measuring emission spectra in (A) and (B), relative fluorescence intensity (FI) by diluting cAMP from 10 to 1 μM cAMP was calculated from (F_10 μM cAMP_×F_A_)/(F_0_×F_B_).

**Response kinetics of Pink Flamindo to cAMP *in vitro***

Fluorescence intensity of purified Pink Flamindo protein at 595 nm was measured in the fluorescence spectrophotometer at every 0.5 sec. Basal fluorescence intensity, normalised to 1, was calculated as the average fluorescence intensity during 10 sec immediately before the application of cAMP.

**Comparison of basal brightness and photobleach curves between Pink Flamindo and mCherry**

HeLa cells were transfected with Pink Flamindo or mCherry in the pcDNA3.1(-) vector as described in the main text, and cultured at either 32°C or 37°C for two days. Imaging was performed as described in the main text by using an objective lens (UPlanApo, 20×, NA = 0.70, Olympus). To obtain photobleach curves, cells were constantly exposed to 2.6W/cm^2^ of green light from a xenon lamp, and images were acquired at every 0.5 sec for 10 min. Cells with similar fluorescence intensities were chosen for data analysis, and maximum fluorescence intensity was normalised to 100% in each cell.

**pH correction in live cell imaging by co-expressing Pink Flamindo and pHluorin**

HeLa cells were transfected with Pink Flamindo and pHluorin, and imaged as described in the main text. After application of 100 μM Fsk, cells were perfused with high K^+^/nigericin solution (130 mM KCl, 10 mM NaCl, 2 mM CaCl_2_, 1 mM MgCl_2_, 10 mM MOPS, 5 μg/mL nigericin; pH 6.4, 6.8, 7.4, 7.6, 7.8) according to previous study ^1^. pH dependency curves of Pink Flamindo and pHluorin were fitted with two exponential curves (pH 6.4 to 7.4, and pH 7.4 to 7.8). Based on the pH estimation calculated from pHluorin signals, effect of pH changes on the FI of Pink Flamindo (F_pH_) was estimated. Corrected FI was calculated from F/F_pH_.

**References**

1. Berg, J., Hung, Y. P. & Yellen, G. A genetically encoded fluorescent reporter of ATP:ADP ratio*. Nat. Metho*d**s** 6, 161–166 (2009).
